# Crohn’s and Colitis Canada’s 2021 Impact of COVID-19 and Inflammatory Bowel Disease in Canada: Mental Health and Quality of Life

**DOI:** 10.1093/jcag/gwab031

**Published:** 2021-11-05

**Authors:** Lesley A Graff, Sharyle Fowler, Jennifer L Jones, Eric I Benchimol, Alain Bitton, James Guoxian Huang, M Ellen Kuenzig, Gilaad G Kaplan, Kate Lee, Mariam S Mukhtar, Parul Tandon, Laura E Targownik, Joseph W Windsor, Charles N Bernstein

**Affiliations:** 1 Department of Clinical Health Psychology, Max Rady College of Medicine, Rady Faculty of Health Sciences, University of Manitoba, Winnipeg, Manitoba, Canada; 2 University of Manitoba IBD Clinical and Research Centre, Winnipeg, Manitoba, Canada; 3 Department of Medicine, University of Saskatchewan, Saskatoon, Saskatchewan, Canada; 4 Department of Medicine, Dalhousie University, Halifax, Nova Scotia, Canada; 5 SickKids Inflammatory Bowel Disease Centre, Division of Gastroenterology, Hepatology and Nutrition, The Hospital for Sick Children, Toronto, Ontario, Canada; 6 Child Health Evaluative Sciences, SickKids Research Institute, Toronto, Ontario, Canada; 7 ICES, Toronto, Ontario, Canada; 8 Department of Paediatrics and Institute of Health Policy, Management and Evaluation, University of Toronto, Toronto, Ontario, Canada; 9 Department of Medicine, McGill University Health Centre, McGill University, Montreal, Quebec, Canada; 10 Department of Medicine, University of Calgary, Calgary, Alberta, Canada; 11 Department of Community Health Sciences, University of Calgary, Calgary, Alberta, Canada; 12 Crohn’s and Colitis Canada, Toronto, Ontario, Canada; 13 Department of Internal Medicine, King Abdulaziz University Hospital, Jeddah, Saudi Arabia; 14 Division of Gastroenterology and Hepatology, Mount Sinai Hospital, University of Toronto, Toronto, Ontario, Canada; 15 Department of Internal Medicine, Max Rady College of Medicine, Rady Faculty of Health Sciences, University of Manitoba, Winnipeg, Manitoba, Canada

**Keywords:** Coronavirus, Crohn’s disease, Mental health, Quality of life, SARS-CoV-2, Ulcerative colitis

## Abstract

There has been a dramatic rise in mental health difficulties during the coronavirus disease 2019 (COVID-19) pandemic. While young adults have the lowest risk of hospitalization and mortality due to COVID-19, they have been identified as being at highest risk of detrimental mental health outcomes during the pandemic, along with women, those with lower socioeconomic status and those with pre-existing mental health conditions. Somewhat of a crisis in mental health has emerged across the general population through the evolution of the pandemic. A national Canadian survey identified a quadrupling of those experiencing pervasive elevated anxiety symptoms early in the pandemic compared to pre-pandemic levels, and a doubling of those with pervasive elevated depressive symptoms. Independent of the pandemic, persons with inflammatory bowel disease (IBD) can face multiple challenges related to their disease, which can result in a significant psychosocial burden and psychologic distress. Anxiety and depression have been found to be more prevalent in persons with IBD. Many potential factors contribute to the increased psychologic distress and negative impacts on mental health of the COVID-19 pandemic on persons with IBD. These include the fears of contracting COVID-19 or infecting other people. Many believe that IBD or its treatments predispose them to an increased risk of COVID-19 or a worse outcome if acquired. Concerns about access to health care add to mental distress. People with IBD generally report lower quality of life (QOL) compared to community controls. Psychologic interventions, in addition to adequate disease control, have been shown to improve health-related QOL. Uncertainty is another factor associated with reduced health-related QOL. Most studies suggest that persons with IBD have suffered QOL impairment during the pandemic in comparison to the pre-pandemic period. Uncertainties brought on by the pandemic are important contributors for some of the reduction in QOL.

**Key Messages**

- Persons with IBD are at increased risk of having mental health disorders, and the presence of mental health disorders can adversely impact the course of IBD.
- During the COVID-19 pandemic, the general population has experienced an increase in distress and mental health disorders.
- Several factors contribute to mental health disorders during the pandemic, including uncertainty regarding risks for morbidity and mortality if COVID-19 is acquired, and uncertainty as to prevention optimization, social isolation and financial stressors from workplace changes.
- The stresses and uncertainties are exacerbated in persons with a chronic disease, such as IBD, since there are added concerns regarding physical health issues, use of immunomodulating therapies and access to health care.
- The COVID-19 pandemic has had a significant negative impact on the quality of life of persons with IBD.

## MENTAL HEALTH IN THE GENERAL POPULATION DURING THE PANDEMIC

The severe acute respiratory syndrome coronavirus 2 (SARS-CoV-2) outbreak has had widespread health, economic and social impact. Health care systems have been challenged to manage an onslaught of severely ill individuals; deaths due to the coronavirus disease 2019 (COVID-19) are now in the millions worldwide; and governments have imposed restrictive measures, including lockdowns and business closures to slow transmission. With pandemic sequelae of uncertainty, fear, social isolation, loss and financial insecurity also being major risk factors for deteriorating mental health (1), it is anticipated that the mental health effects of the pandemic will last well beyond the acute medical consequences (2,3).

Evaluations of population-level mental health early in the pandemic signaled high levels of distress across multiple countries (4–7). Substance use rates, and particularly alcohol consumption, also increased (8,9). While many early studies used convenience samples to take a rapid mental health pulse, potentially biasing outcomes (10), subsequent studies using representative probability samples confirmed initial assessments. A national Canadian survey identified a quadrupling of those experiencing pervasive elevated anxiety symptoms early in the pandemic compared to pre-pandemic levels (5% compared to 20% as of April, 2020) and a doubling of those with pervasive elevated depressive symptoms (4% compared to 10%) (11).

Assessing population distress over time, the Canadian survey found these high levels of anxiety and depression symptoms had not subsided by December 2020 (12) and, in the most recent national poll in February 2021, were at their highest levels (anxiety: 25%; depression: 17%) (13). Population prevalence of clinically significant distress in the United Kingdom, measured through their national longitudinal household survey, identified a significant escalation from 2018 to 2019 levels of 19.4% to 30.6% in April 2020—well above any upward trends predicted from prior years’ trajectories (8), which persisted over the next months (14). This study, which sampled the same households, found some indication of population adaptation, with levels of clinically significant distress settling back to pre-pandemic levels in the fall, 2020 measurement (15). The national longitudinal household survey for the United States (also using a probability-based representative sample comparing participants over time) showed a similar trend with early pandemic distress rates significantly higher than pre-pandemic, and some settling of these rates a few months into the pandemic (16). These positive adjustments were associated with changes in perception of risk for acquiring COVID-19 or being financially impacted (16). While evidence of population adaptation to the pandemic challenges are encouraging, with the pandemic not yet contained (as of spring 2021), the trajectory for mental health impact is unclear, and pronounced distress levels may continue to fluctuate.

In addition to general distress levels, serious outcomes escalated from pre-pandemic levels. A large U.S. epidemiologic study, based on 6 million emergency department visits that involved mental health presentations, compared visits during pandemic months in 2020 to the same period in 2019 and found visit counts were significantly higher in 2020 for suicide attempts and overdoses (17). Further, the mental health impact of the COVID-19 pandemic has not been uniform. While young adults have the lowest risk of hospitalization and mortality due to COVID-19, they have been identified as being at highest risk of detrimental mental health outcomes during the pandemic, along with women, those with lower socioeconomic status and those with pre-existing mental health conditions (6,9,14,18–20). Even outside the context of the pandemic, these groups have elevated risk for mental health concerns; however, during the pandemic, there has also been a disproportionate economic impact on these demographic groups, who tend to be overrepresented in lower wage jobs and retail and hospitality sectors, which were hard hit by pandemic restrictions.

Experience from prior severe respiratory disease outbreaks has suggested mental health concerns may be particularly prominent for persons who contracted the disease. A meta-analysis of studies that included data related to SARS, Middle East respiratory syndrome and COVID-19 found high point prevalence rates post-illness for post-traumatic stress disorder (32%) as well as depression and anxiety (14%), with mental illness post-SARS persisting up to 4 years (21). An epidemiologic study in the United States reviewing over 62 million health records matched 62,000 COVID-19-positive cases with cases of other types of illnesses; the study found that individuals with COVID-19 had more than double the risk of being newly diagnosed with a mental health disorder (i.e., no prior history) in the weeks following the COVID-19-positive diagnosis, compared to the non-COVID illness cohorts (22). Reviewing mental health outcomes over a 6-month period for more than 236,000 persons with a COVID-19-positive diagnosis, they found that one in three (33% incidence rate) COVID-19-positive persons had neurological or psychiatric diagnoses, with anxiety disorder (17%) being most common (23).

## MENTAL HEALTH IN PERSONS WITH INFLAMMATORY BOWEL DISEASE DURING THE PANDEMIC

Persons with inflammatory bowel disease (IBD) can face multiple challenges related to their disease, including the chronic nature of the disease, its incurability, unpredictability and severity of symptoms, as well as concerns regarding disease and treatment complications; this can result in a significant psychosocial burden and psychologic distress (24). Anxiety and depression have been found to be more prevalent in persons with IBD compared to healthy controls, with rates of anxiety and depression ranging from 19% to 28% during remission and 34% to 66% during active flares (25). Untreated mental health disorders have been associated with poor IBD outcomes including more severe IBD symptoms and more frequent flares (26,27), poor medication adherence (28), higher hospitalization rates (29) and increased health care costs (30).

Many potential factors could contribute to the increased psychologic distress and negative impacts on mental health of persons with IBD related to the COVID-19 pandemic. D’Amico et al. sought to elicit the patients’ points of view to investigate the concerns, fears and behaviors of people with IBD during the early phase of the pandemic (31). An anonymous web survey was conducted and included 3815 participants from 51 countries around the world. Most respondents feared contracting COVID-19 (3242, 85%) or infecting other people (3330, 87%). Just under a third of respondents believed that IBD predisposed them to an increased risk of COVID-19 (1150, 30%) and nearly two-thirds believed that immunosuppressive drugs were associated with a higher risk of infection (2427, 64%). Similar concerns were identified in a study from China, in which 2277 people with IBD were surveyed (32). Respondents were worried about the risk for SARS-CoV-2 infection for themselves and their family (58%) and more than half were concerned about the difficulty in seeing physicians (52%). This study also assessed psychosocial impacts of the pandemic and found that more than 50% of participants reported some degree of mood changes, most commonly reporting moderate-to-severe psychologic change in the middle of the outbreak in China (i.e., mid-February 2020). Most respondents (77%) did not change IBD medications during the outbreak. However, of those with a change, the main reasons were recommendations from their treating physician (30%), being unable to receive intravenous infusions (28%) and the availability of physicians or facilities (28%). Despite this, the majority of respondents reported their disease was stable (74% during the initial outbreak from January to March 2020, and 81% in the later phase in mid-April 2020).

Harris et al. demonstrated similar findings in a survey of 685 people with IBD in the United Kingdom (33). In this study, 37% of respondents reported a flare in their IBD symptoms and 87% reported their medications had remained unchanged throughout the lockdown. Over 50% of participants reported a negative or very negative impact of the pandemic on their quality of life (QOL). Anxiety or depression were the most common comorbidities, self-reported by 14.9% of participants, and were correlated with a greater stress score at all phases in the pandemic. A multi-nation survey of 243 individuals with Crohn’s disease also found that an increase in active disease symptoms was associated with increased perceived stress (34). This study used an anonymous survey to retrospectively assess symptoms of Crohn’s disease in the months prior to COVID-19 (January and early February 2020) and again during the early stages of the pandemic (March and April 2020). The Manitoba Inflammatory Bowel Disease Index was used as a measure of disease activity (35). A total of 17% (40/243) of respondents reported a change from inactive to active Crohn’s disease symptoms with a 25% relative increase in active symptoms between the pre-COVID-19 period compared to the COVID-19 period (*P* < 0.001). The most frequently reported reason for a change in symptoms was increased stress and/or feeling overwhelmed (50%). The relative percent increase in active symptoms was more pronounced (42%) among those reporting current stress (*P* < 0.001).

Mosli et al. further assessed the psychologic impact of COVID-19 on people with IBD in Saudi Arabia through a validated Arabic version of the Hospital Anxiety and Depression Scale (36). Of the 1156 persons with IBD assessed, normal, borderline and Hospital Anxiety and Depression Scale–Anxiety subscale scores consistent with a diagnosis of anxiety were reported by 36.6%, 15.1%, and 48.4% of participants, respectively. With respect to depression, 69% of participants had normal scores, 30.1% had borderline scores and no participants reported scores consistent with depression, which would be atypical for most other IBD cohorts. For instance, Trindade et al. surveyed 124 persons with IBD from Portugal (37). Most participants (51.6%) presented normal (non-clinically significant) levels of depressive symptoms; 27.4% presented with mild severity, 16.10% moderate and 4.8% severe. However, with respect to anxiety, 29.8% and 18.5% presented normal and mild anxiety levels, respectively; about half presented with moderate (37.1%) and severe (14.5%) anxiety.

Finally, Castellini et al. conducted a survey aimed to explore the role of participants’ psychologic predispositions in dealing with the COVID-19 pandemic using the Patient Health Engagement Scale (38). This study showed that people with higher scores on the Patient Health Engagement scale had significantly lower levels of the perceived risk of COVID-19 emergency; they also experienced lower levels of stress and higher levels of coping self-efficacy. Increased health engagement seemed to mitigate negative reactions to the COVID-19 pandemic.

## QOL IN PERSONS WITH IBD

QOL is a broad, multidimensional concept that usually includes subjective evaluations of both positive and negative aspects of life (39,40). The Centres for Disease Control and Prevention has defined QOL as ‘an individual’s or group’s perceived physical and mental health over time’ (39). The concept of health-related quality of life (HRQOL) and its determinants has evolved since the 1980s to encompass aspects of overall QOL that have been shown to affect either physical or mental health (39,41,42). HRQOL questions have become an important component of public health surveillance and are considered valid future indicators of unmet needs and intervention outcomes. Self-assessed health status is a more powerful indicator of mortality and morbidity than many objective measures of health (43,44).

### HRQOL in Persons Living With IBD Pre-Pandemic

HRQOL is impaired at some point in every person with IBD, and many live with chronically impaired HRQOL. IBD affects young individuals at a time in their lives when they are most likely to be pursuing major employment, family building and personal milestones of critical importance. The pursuit of such critical milestones is often impeded by the unrelenting and debilitating symptoms and the psychologic distress associated with IBD (45–48). Measuring and evaluating the disability associated with IBD, and the impact of IBD on a person’s QOL, is paramount to understanding the often hidden burden of this disease for persons living with IBD and for society as a whole. On balance, data from most population-based studies suggest that persons living with IBD have significantly lower HRQOL when compared to that of the general population; this is particularly true for those with more severe disease activity (49–55), which remains the most significant predictor of physical and mental HRQOL (56). QOL domains most impacted in adult and pediatric persons with IBD are general health, mental health and social functioning. Youth with IBD have lower HRQOL compared with their healthy peers and higher levels of impaired school-related HRQOL than healthy or chronically ill youth (57–60). Youth-reported reductions in HRQOL have been associated with greater health care resource use, including IBD-related hospital admissions, emergency department visits, use of psychologic services, telephone calls to clinicians, gastroenterology clinic visits and referral to pain management (61). Lower HRQOL has been associated with lower psychosocial functioning and school functioning among youth with IBD in comparison to both healthy comparator groups chronic illness comparator groups (60).

Several factors reduce QOL for persons living with IBD (24,49–58,62). Psychologic interventions, in addition to adequate disease control, have been shown to improve HRQOL (see Table 1 for a summary of factors that influence HRQOL) (63–74). Uncertainty is another factor associated with reduced HRQOL (75). Health information gathering through the internet by persons with Crohn’s disease has been associated with reduced certainty (75). Therefore, people living with IBD require access to providers with the ability to screen for, and manage, psychological distress as part of a transdisciplinary care approach.

**Table 1.** Factors that influence QOL in people living with IBD

	Impact on QOL		Mechanism of influence on QOL
	Children	Adults	
IBD disease activity	Negative	Negative	• Self-esteem • Relationships • Hygiene • Security • Depressive symptoms • Social isolation • Psychologic distress
Psychologic distress	Negative	Negative	• Reduced energy • Impaired body image • Maladaptive coping mechanisms • Social isolation
Psychologic interventions	Positive	Positive	• Reduction of exacerbating factors related to increased disease-related psychologic distress
Surgery	Negative	Negative	• Fear of complications • Need for an ostomy • Body image • More severe form of disease
Pain	Negative	Unknown	• Disability • Depressive symptoms • Disease-related psychologic distress
Parental stress	Negative	Unknown	• Perceived difficulty of medical stressors
Effective medical therapy	Positive	Positive	• Induction of long-term remission
Health information gathering from internet	Negative	Negative	• Reduced certainty
Poor sleep quality	Negative	Negative	• Fatigue • Daytime dysfunction

IBD, Inflammatory bowel disease; QOL, Quality of life.

### HRQOL for Persons Living With IBD During the Pandemic

The global community was launched into an extended and ongoing period of uncertainty with the onset of the COVID-19 pandemic. Those living with chronic, immune-mediated diseases were suspected to have the largest reductions in QOL given significant uncertainty relating to the morbidity and mortality of SARS-CoV-2 infection, as well as the relative impact of various immunosuppressive therapies on COVID-19 disease course, and the social isolation and financial hardships of public health restrictions imposed to control population transmission of SARS-CoV-2. Exposure to rapidly changing health information from both reliable and unreliable sources, reductions in access to health care providers and health care resources, as well as changes in how IBD care was being delivered were also potential sources of uncertainty and psychologic distress.

Given the known negative effects of uncertainty and increased psychologic distress on QOL, there has been interest in better understanding how the global COVID-19 pandemic has impacted QOL in persons living with IBD. Most studies suggest impairment of QOL during the pandemic in comparison to the pre-pandemic period (33,76–79). Grunert et al. conducted a cross-sectional survey of IBD practices in Germany in order to better understand the impact of the pandemic on daily life for adults living with IBD (77). They observed increased fears of infection, hospitalization and going to public spaces—including hospitals and clinics for biologic infusions; these concerns were heightened if they were taking immunosuppressive therapies. As a result, participants responded that they were less likely to leave home compared to their peers. Another UK survey was performed with moderate- and high-risk IBD populations (IBD populations deemed to be at high risk of morbidity from COVID-19) (33). Out of 685 respondents, the majority reported a negative impact of the pandemic on QOL as well as significant increases in perceived stress. Many respondents identified remote health care delivery as a mechanism through which to alleviate this stress. Prospectively conducted interviews of 67 people with IBD in which patient-reported outcomes were measured suggested that persons with active disease experienced significant reductions in QOL domains (*P* = 0.01) and emotional domains (*P* = 0.04) (79).

## CONCLUSIONS

IBD negatively influences HRQOL and introduces disability that can impair daily activities, thus affecting interpersonal relationships, life activities, social participation and mental well-being. HRQOL is uniquely impacted in children and adolescents with IBD. Moreover, the entire family can suffer collectively from reduced HRQOL as parental stress is commonly experienced. The SARS-CoV-2 pandemic has been observed to further impair QOL making access to supports and health system interventions to alleviate those factors that are the key drivers of reductions in QOL critically important; however, this proved to be a challenge, even in the pre-pandemic era. These facts highlight the importance of accurate, timely and reliable information; access to mental health supports; limitations in disruption to care and medical therapies; as well as ongoing, rapid research and patient-centered knowledge translation as we make advances in our understanding of COVID-19 disease course in those with IBD as well as the effectiveness and safety of COVID-19 vaccines in persons living with IBD.
